# The Portuguese version of the Screen for Disordered Eating: Validity and reliability in middle aged and older women

**DOI:** 10.1192/j.eurpsy.2023.912

**Published:** 2023-07-19

**Authors:** A. T. Pereira, M. J. Brito, R. V. Duarte, C. Marques, D. Pereira, C. Cabaços, A. Macedo

**Affiliations:** 1 Institute of Medical Psychology, Faculty of Medicine, University of Coimbra; 2 Coimbra Institute for Biomedical Imaging and Translational Research; 3 Coimbra University Hospital Centre; 4 Faculty of Medicine, University of Coimbra; 5 Department of Psychiatry, Coimbra University Hospital Centre, Coimbra, Portugal

## Abstract

**Introduction:**

Besides the traditionally studied group of young females, disordered eating occurs in all age groups (Eedena, Hoekena, and Hoek 2021). In recent years, there has been an increase in the prevalence of eating disorders and symptoms in middle-aged and older women (40 years old and over) (Mangweth-Matzek and Hoek 2017).

Experts in eating psychopathology in special groups such as Samuels, Maine and Taltillo (2019) suggest the use of the Screen for Disordered Eating (SDE; Magen et al. 2018) in the psychometric assessment of women in middle age and older. The SDE was developed to allow the Eating Disorders (ED) screening in Primary Health Care in people of all ages and without excluding Binge Eating Disorder (BED).

The SDE is composed of five items (yes or no answers), extracted from other validated self-reported questionnaires for the assessment of eating psychopathology.

**Objectives:**

To analyze the psychometric properties of the Portuguese Version of the Screen for Disordered Eating in a sample of women from the general population aged 40 and over.

**Methods:**

Participants were 516 women with a mean age of 50.28 of years old (± 8.063; range: 40-80). They answered an online survey including the preliminary Portuguese version of the SDE and the *Portuguese version of the* Eating Disorder Examination – Questionnaire (EDE-Q-7; Pereira et al. 2021).

**Results:**

Confirmatory Factor Analysis showed that the unidimensional model presented good fit indexes (χ^2^/df=1.502; RMSEA=.0311, p<.001; CFI=.987 TLI=.995, GFI=.965). The Cronbach’s alfa was .762. All the items contributed to the internal consistency, as they presented item-total correlations above .40 and the exclusion of each one would decrease the alpha. Pearson correlations between SDE and the EDE-Q-7 were significant (p<.01), positive and moderate/high, as follows: .516 with the total score and .318, .503 and .536 respectively with the dimensional scores of Dietary restraint, Shape/weight overvaluation and Body dissatisfaction.

**Conclusions:**

As observed with the original English-language scale, the Portuguese version of the SDE has shown good validity (construct and concurrent) and internal consistency. As such, the SDE might be a useful tool to investigate disordered eating psychopathology in older women. In the near future we will determine the SDE cut-offs with the best combination of sensitivity and specificity to screen for eating disorders in this populational group.

**Disclosure of Interest:**

None Declared

